# Ancillary Testing in Lung Cancer Diagnosis

**DOI:** 10.1155/2012/249082

**Published:** 2012-01-04

**Authors:** William Dubinski, Natasha B. Leighl, Ming-Sound Tsao, David M. Hwang

**Affiliations:** ^1^Department of Pathology, Toronto General Hospital, University Health Network, 200 Elizabeth Street, Toronto, ON, Canada M5G 2C4; ^2^Department of Laboratory Medicine and Pathobiology, University of Toronto, 1 King's College Circle, Toronto, ON, Canada M5S 1A8; ^3^Department of Medical Oncology and Hematology, Princess Margaret Hospital, University Health Network, 610 University Avenue, Toronto, ON, Canada M5G 2M9

## Abstract

The pathologic diagnosis of lung cancer historically has relied primarily on morphologic features of tumors in histologic sections. With the emergence of new targeted therapies, the pathologist is called upon increasingly to provide not only accurate typing of lung cancers, but also to provide prognostic and predictive information, based on a growing number of ancillary tests, that may have significant impact on patient management. This review provides an overview of ancillary tests currently used in the pathologic diagnosis of lung cancer, with a focus on immunohistochemistry and molecular diagnostics.

## 1. Introduction

Primary lung cancer has been classified historically into two clinically relevant groups: small cell lung cancer (SCLC) and nonsmall cell lung cancer (NSCLC). This distinction was clinically useful as available treatment strategies differed significantly between these two groups. In recent years, the emerging evidence of differential response to new targeted therapies and the identification of molecular differences between specific subtypes of NSCLC increasingly necessitate greater accuracy in the subtyping of NSCLC.

The current WHO classification of lung cancer [1] has been based almost entirely by assessment of morphologic features using standard hematoxylin and eosin (H&E) stained sections of tumors. However, a growing number of ancillary studies can help with classification, such as the use of immunohistochemistry (IHC). Beyond simple classification, however, ancillary testing for molecular aberrations is entering routine practice and delivers additional prognostic and predictive information. A new multidisciplinary classification system for primary lung adenocarcinomas has emerged recently [2]. While this system is still based largely on morphology, it moves towards incorporating recent advances in clinical and molecular medicine. In this review, we summarize ancillary tests currently used in the pathologic diagnosis of lung cancer, with a focus on immunohistochemistry and molecular diagnostics.

## 2. Immunohistochemistry

Immunohistochemistry involves the detection and localization of antigens or proteins in tissue sections by the use of antibodies that bind specifically to the antigen of interest. The antibodies are coupled to a detection system which allows them to be visualized in tissue sections. IHC has a range of applications in the practice of pathology and is commonly used by pathologists to help in distinguishing cell types or their origin, using markers that are expressed differentially between different cell types and organs. Additionally, IHC enables one to observe or determine the localization and distribution of various antigens or proteins within the tissue.

However, it needs to be recognized that IHC is neither 100% specific nor 100% sensitive. For example, thyroid transcription factor-1 (TTF-1) is widely known as a marker for pulmonary adenocarcinoma but is also highly expressed in thyroid tumors and may uncommonly also be expressed in carcinomas originating from other primary sites, including, for example, colorectal carcinoma [3]. Morphologic features on H&E sections remain the basis for diagnosis, and ancillary tests need to be interpreted in the context of the histomorphologic findings. Despite these limitations, it has been shown that a high degree of accuracy in the subtyping of NSCLC can be achieved by applying a simple panel of immunohistochemical markers including TTF-1, tumor protein 63 (P63), cytokeratin (CK) 7, and CK5/6 [4, 5]. Other antibodies such as desmocollin-3 and napsin A have also been recently described as helpful in further refining the subtypes in difficult cases [6, 7].

CKs are intermediate filament proteins that provide structural support within the cytoplasm of epithelial cells. Fifty-four CK genes have been identified, and the corresponding keratin proteins are classified by molecular weight and isoelectric pH [8]. In the most general terms, tumors of epithelial origin (termed carcinomas or adenocarcinomas, if gland forming) express CKs, which differentiates them from tumors of mesenchymal origin (sarcomas), hematopoietic origin (lymphomas/leukemias), and melanoma. The various epithelial tissues of the human body also express different CKs, thus different epithelial tumors may often be distinguished based on their unique cytokeratin expression profile.

TTF-1 plays an important role in the embryogenesis of lung, and its expression remains high in type II pneumocytes and Clara cells [9]. TTF-1 has been used as an immunohistochemical marker for primary lung adenocarcinoma, despite recent reports of occasional aberrant TTF-1 staining in tumors from other primary sites (e.g., [3]). TTF-1 is known to regulate the expression of several lung-specific proteins including napsin A, surfactant proteins, and others. Antibodies to napsin A in combination with TTF-1 have been proposed as additional evidence of pulmonary origin of a tumor. 

### 2.1. Squamous Cell Carcinoma

In H&E stained sections, squamous differentiation is identified by keratinisation and/or formation of intercellular bridges. Both features are specific for squamous cell differentiation and are not seen in other tumor types. While these features are readily observed in well-differentiated tumors, they may be difficult to appreciate or absent in poorly differentiated tumors, especially in small biopsy samples or fine needle aspirate cytology specimens. In such cases, an IHC panel including P63, CK5/6, TTF-1, and CK7 may be helpful, with positive staining for P63 and CK5/6 and concurrent lack of staining for TTF-1 and CK7 supporting squamous differentiation (Table 1) [5]. 

From a clinical standpoint, it is important to note that squamous differentiation is not evidence of the tumor's site of origin. Metastatic squamous cell tumors to the lung are histologically and immunohistochemically identical to primary lung squamous tumors. At present, there are no immunohistochemical or molecular markers in routine use that reliably differentiate primary pulmonary from metastatic squamous cell carcinomas, as TTF-1 typically is not expressed in pulmonary squamous cell carcinomas. Therefore, clinical history is crucial to determining the site of origin. 

The accurate diagnosis of squamous cell histology has important therapeutic implications. Certain systemic therapy agents are not used in patients with squamous histology for safety or efficacy concerns. Bevacizumab, a monoclonal antibody directed against vascular endothelial growth factor (VEGF) is associated with an increased risk of life threatening pulmonary hemorrhage in patients with squamous cell histology [10]. The association between bleeding complications and this histological type has been seen with other VEGF inhibitors although not all [11, 12]. Also, pemetrexed, a chemotherapeutic agent has been associated with inferior outcomes compared with docetaxel chemotherapy. By contrast, pemetrexed use in the first-, second-line, and maintenance settings has been associated with superior outcomes in patients with nonsquamous histology [13, 14]. These studies confirm that accurate classification of NSCLC has an important role in patient management and outcomes. 

### 2.2. Adenocarcinoma

**Figure 1:**
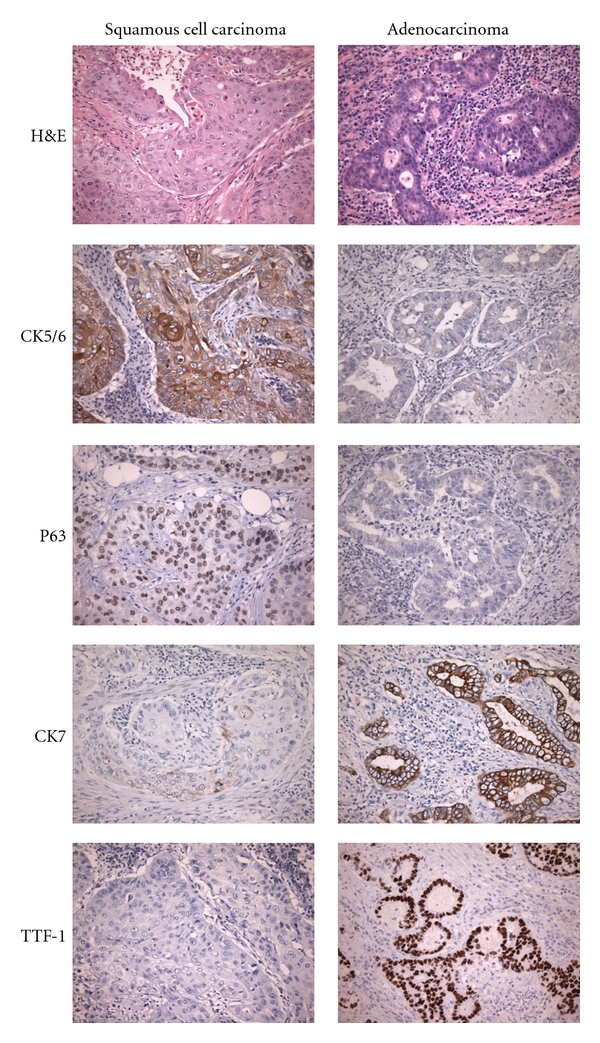
Immunohistochemistry stains in squamous cell carcinoma and adenocarcinoma of lung. H&E: hematoxylin and eosin; CK: cytokeratin; TTF-1: thyroid transcription factor 1. Squamous carcinomas are typically positive for CK5/6 and P63, and negative for CK7 and TTF-1, with the reverse profile for adenocarcinoma although this case of squamous cell carcinoma demonstrates focal weak staining for CK7.

The WHO classification [1] of lung tumors has long divided adenocarcinomas primarily into acinar, papillary, solid, bronchioloalveolar (BAC), or mixed subtypes based on histomorphologic features. Classically, adenocarcinomas display gland formation on H&E, although subtypes with bronchioloalveolar or solid patterns of growth may lack well-defined glandular structures. The BAC subtype (or adenocarcinoma in situ (AIS) in the new IASLC/ATS/ERS classification [2]) is characterized by exclusively “lepidic” growth pattern, in which neoplastic cells grow along the surfaces of preexisting alveolar structures, without evidence of invasion. Two variants of BAC have been recognized classically, mucinous and nonmucinous. The mucinous variant (mucinous AIS or invasive mucinous adenocarcinoma in the IASLC/ATS/ERS classification [2]) is composed of tall columnar cells with abundant pale cytoplasm that stains positively with histochemical stains for mucin, such as mucicarmine or periodic acid Schiff (PAS). Pulmonary adenocarcinoma is differentiated from squamous cell carcinoma by being typically positive for CK7 and TTF-1, and negative for p63 and CK5/6 (Figure 1). Although most pulmonary adenocarcinomas are positive for TTF-1, a significant subset is negative (15–30%), especially the mucinous BAC subtype, or those originating in more central locations [15]. 

Primary lung adenocarcinoma must also be differentiated from adenocarcinoma that has metastasized to lung. Clinical history and IHC can be invaluable in this regard. In particular, the differential expression of CK7 and CK20 may be very useful in characterizing the origin of epithelial neoplasms. Pulmonary adenocarcinoma is typically CK7+/CK20− by IHC although this cytokeratin profile is not specific to adenocarcinomas of lung and may be seen also in tumors arising from the breast, thyroid, upper gastrointestinal and pancreaticobiliary tracts, and gynecologic tract. Tumors demonstrating CK7−/CK20+ staining profile include colorectal and Merkel cell carcinomas. CK20 staining may occasionally be seen in pulmonary adenocarcinomas, but CK7 typically is also positive in such cases. While an exhaustive discussion is beyond the scope of this manuscript, suffice to indicate that most primary lung adenocarcinomas are CK7 and TTF-1 positive, although as previously noted, TTF-1 staining may also be seen in thyroid tumors and infrequently in tumors originating from other body sites. Lack of TTF-1 expression does not exclude pulmonary origin for a CK7-positive adenocarcinoma in the lung; however, in this situation, metastatic carcinoma arising from other body sites that demonstrate a CK7+/CK20− cytokeratin profile would need to be excluded clinically. 

Napsin A, an aspartic acid protease whose expression in the lung is regulated by TTF-1, has also shown promise in helping to differentiate primary lung from metastatic adenocarcinomas. While napsin A expression may also be seen in normal kidney and in a proportion of renal tumors, positivity for both TTF-1 and napsin A is a strong indication that an adenocarcinoma originated from lung [6, 16]. 

### 2.3. Neuroendocrine Tumors

Tumors with neuroendocrine (NE) differentiation include small cell carcinoma, large cell neuroendocrine carcinoma, and typical and atypical carcinoid tumors. NE tumors are defined ultrastructurally by having neurosecretory granules and immunohistochemically by positivity for NE markers (Table 2). According to a recent international workshop on pulmonary NE tumors, CD56 has a sensitivity of 95% and a specificity of 97% for detecting cells showing NE differentiation. The sensitivity for synaptophysin and chromogranin is 80–85%, and the specificity for both is 97% [17]. Using a panel of two or three NE markers ensures a high degree of accuracy for detecting NE differentiation. 

Differentiating between the different types of NE tumors may be very difficult in small biopsy specimens because the small sample may not be entirely representative of the overall tumor and is subject to crush artefact because of the relative fragility of these cells. In some cases, the evaluation of Ki-67 may be useful in this regard. Ki-67 is a marker of cell proliferation, and a recent review has concluded that the Ki-67 proliferation index of typical carcinoid (TC) is less than 2%, while atypical carcinoid (AC) is less than 20% (typically around 10%) [18]. Small cell carcinomas often have a Ki-67 proliferation index of greater than 60%. It has been suggested that a Ki-67 index of less than 25% excludes small cell carcinoma [19]. 

Differentiating primary NE tumors of the lung from NE tumors from other body sites may also be a challenge, as NE tumors arising from other body sites may demonstrate identical morphology. In this regard, expression of TTF-1 may be helpful to identify a carcinoid tumor of lung origin, as carcinoid tumors from other sites rarely express this marker [20]. However, small cell carcinomas arising from the lung frequently express TTF-1 while those arising from other sites may occasionally also express TTF-1. In the case of small cell carcinoma with TTF-1 positivity, lung origin is favored, but this is not entirely definitive [21]. 

### 2.4. Other IHC Markers

**P53**

P53 functions as a tumor suppressor protein by playing a key role in cell cycle progression, apoptosis, and DNA repair [22]. Overexpression of P53 in NSCLC has been reported to be both a prognostic marker, associated with increased tumor aggressiveness and shorter overall survival, and a potential predictive marker, associated with a favorable response to platinum-based adjuvant chemotherapy, resulting in a survival benefit [23]. However, this needs additional validation.

**ASH1**

From an embryologic perspective, there is now evidence that the transcription factor achaete-scute homologue-1 (ASH1) is pivotal for NE cell differentiation and may be necessary for transformation to NE lung carcinoma [24]. Immunohistochemical analysis of ASH1 is available but not routinely used outside of the research setting at present. It may potentially help to identify early progenitor cells that are committed to NE differentiation and better define subsets of lung cancers demonstrating NE differentiation [24].

## 3. Molecular Diagnostics in NSCLC

Molecular testing of lung cancers has seen significant advances in recent years. The potential information derived from molecular tests is enormous and has increasing applications in clinical settings. In the setting of lung cancer, molecular techniques currently employed include both polymerase chain reaction- (PCR) based tests and fluorescent in-situ hybridization (FISH). Both of these tests can be done using formalin-fixed-paraffin-embedded (FFPE) tissue provided that there is sufficient tumor quantity and a nondegraded specimen. PCR is a molecular technique used to amplify short segments of DNA. The amplified DNA from tumor samples can then be subject to various analyses to identify differences (mutations) from a “normal” control. FISH is a technique whereby fluorescent labeled probes are admixed with a test sample and the probes bind to specific DNA sequences if present. Fluorescent microscopy is then used to analyze if and where on the chromosomes the probes have bound. Probes can be designed to bind either normal segments of DNA or segments with known aberrations. A brief review of selected genetic abnormalities with clinical relevance in lung cancer follows (summarized in Table 3). A more extensive review of molecular predictive and prognostic markers has been published elsewhere [25]. 

### 3.1. Epidermal Growth Factor Receptor (EGFR)

EGFR represents a family of transmembrane tyrosine kinase growth factor receptors involved in a wide array of cellular processes, including proliferation, apoptosis, angiogenesis, and others. A number of patients with NSCLC have dysregulated EGFR resulting in overexpression, amplification, or mutations [26]. Clinical and pathological features associated with high-frequency mutations in the EGFR tyrosine kinase (TK) domain in NSCLC include East Asian ethnicity, females, light/never smokers, and tumors with adenocarcinoma histology. In the general North American or European population, approximately 10–17% of NSCLC cases may have EGFR mutations [27]. In light or never-smoking Asian patients with adenocarcinoma, the rate of EGFR mutations may be as high as 60% [28]. Mutated EGFR predicts for high response rate to EGFR TK inhibitor (TKI) therapy, a first-line treatment choice in advanced NSCLC patient population [29], with better progression-free survival and quality of life. Accurate mutation analysis is also important because progression-free survival is shorter if EGFR wild-type patients are treated with EGFR TKIs as opposed to conventional chemotherapy. There is also evidence that different mutations may have differential response rates and relative resistance to EGFR TKI's [30]. 

Aberrations in EGFR are common in a wide variety of human cancers, but the site of mutation varies with the type of malignancy. Activating mutations in EGFR result in constitutively activated downstream signalling pathways. Studies on EGFR mutations in NSCLC show that approximately 90% occur within exons 18–21 [31]. The specific type of mutation may hold predictive relevance in that some mutations confer higher affinity for certain EGFR TKIs and some confer resistance to certain EGFR TKIs, for example, mutations in exon 20 and the T790M mutation. Various different methods are currently used for the detection of EGFR mutations (reviewed in [32]). Of these, direct sequencing of PCR amplicons from the EGFR gene is perhaps the most widely used but is also cost and labor intensive. Targeted detection of specific mutations is also an option, given that two mutations account for more than 90% of all EGFR mutations in NSCLC (exon 19 deletions and the exon 21 L858R mutation) [33]. However, the development of monoclonal antibodies directed against the two most common mutations may enable immunohistochemical screening for EGFR mutations, providing rapid results at a fraction of the cost of current molecular testing [34]. 

### 3.2. K-*RAS*

The *ras* family of oncogenes encode for proteins that mediate signalling pathways controlling cell growth. There are three distinct *ras* genes (H-*ras*, K-*ras*, N-*ras*), each of which has been associated with various malignancies. In NSCLC, >90% of *ras* mutations occur on the K-*ras* gene, which would result in the constitutive activation of downstream signalling pathways. K-*ras* mutations are seen almost exclusively in smokers, and this relationship does not seem to be dose or time dependent [35]. K-*ras* mutations in NSCLC occur mainly in adenocarcinoma. As a prognostic marker, K-*ras* mutation appears to be associated weakly with poorer outcome [36]. K-*ras* mutations rarely occur in tumors with EGFR mutations. Their presence has been associated with relative resistance to EGFR TKI therapy [37] although currently patients with K-*ras* mutations are not excluded from EGFR TKI therapy. In the rare patient that has both a K-*ras* mutation and an activating EGFR mutation, EGFR TKI therapy may be appropriate [38, 39]. 

### 3.3. EML4-ALK

The anaplastic lymphoma kinase (ALK) gene was originally identified as part of a chromosomal translocation found in a subset of anaplastic large cell lymphomas. Subsequently, it has been shown that ALK is dysregulated in a number of solid and hematologic malignancies. The mechanisms of dysregulation include translocations as well as mutations in the tyrosine kinase domain [40]. In 2007, a translocation between echinoderm microtubule-associated protein-like 4 (EML4) and ALK was identified in a subset of lung adenocarcinomas [41]. The fusion product has oncogenic properties, and transgenic mice that express EML4-ALK in alveoli develop hundreds of nodules of adenocarcinoma [42]. 

The result of an early-phase trial with crizotinib, an inhibitor developed for ALK and Met, has generated considerable interest as a therapeutic option in patients with NSCLC who harbor EML4-ALK mutations [43]; a number of clinical trials are currently underway to determine their efficacy and parameters for optimal use. 

The overall incidence of EML4-ALK rearrangements in NSCLC appears to be approximately 3.5% [44]. There appears to be a similar frequency of ALK mutations across different ethnicities, suggesting that race may not be as relevant as for EGFR mutations. Clinical parameters associated with ALK mutations include never/light smokers, and in some studies there is an association with younger patient age and male sex [45, 46]. From a histologic perspective, EML4-ALK rearrangement is seen almost exclusively in adenocarcinomas, and only rare cases have been reported in squamous cell carcinoma [41, 46]. Interestingly, signet ring cell histology has been associated frequently with ALK rearranged tumors [45]. 

A variety of techniques are available to detect ALK gene rearrangements including FISH, IHC, and PCR. FISH probes that identify ALK rearrangements are commonly used in clinical trials. The limitation of this method is that the translocation can be difficult to detect due to the small size of the inversion [47]. Attempts to use IHC to detect ALK-rearranged tumors initially met with limited success as the fusion protein is expressed at low levels; the development of novel specific and sensitive antibodies, however, seems on the horizon [48]. PCR-based techniques have not gained widespread use as PCR-based detection of the fusion transcript is generally not optimal in FFPE tissue. The optimal method for identifying EML4-ALK fusion is yet to be definitively determined [49] although FISH is widely viewed as the current gold standard. 

### 3.4. MET

MET is a receptor tyrosine kinase involved in important signalling pathways related to cell proliferation, angiogenesis, and tumor aggression [50]. MET signalling becomes dysregulated in both NSCLC and small cell lung cancer through various mechanisms, including overexpression, mutations, amplifications, and autocrine/paracrine activation of hepatocyte growth factor [51]. MET gene amplification as detected by fluorescence in situ hybridization may be a poor prognostic factor in NSCLC [52]. MET amplification has also been implicated as one mechanism for acquired resistance to EGFR TKI therapy and may be seen in 5 to 20% of resistant cases [53, 54]. As a therapeutic option, MET inhibitors alone or in combination with EGFR TKIs are in clinical trials. 

## 4. Conclusion

Clinical, pathological, and genetic parameters are beginning to converge in NSCLC. Selection of patients based on clinical and histopathologic criteria can, for example, considerably increase the likelihood of identifying EML4-ALK and EGFR mutations. In addition, the development of treatment protocols directed at specific molecularly or histologically defined subsets of NSCLC has improved treatment outcomes for patients. The role of the pathologist is now to provide not only accurate subtyping of lung cancers, but increasingly also to provide prognostic and predictive information that is critical to patient outcomes. Future developments will continue to refine our understanding and approach to lung cancer. Several lung cancer studies have used microarray platforms to measure the expression of thousands of genes simultaneously. The results generated provide unique gene-expression profiles with significant prognostic and predictive impact [55, 56]. Gene expression profiling may also help refine tumor classification. While these and other molecular profiling strategies need further validation in prospective studies, currently available technology points to a future where the molecular profiling will increasingly help guide classification, treatment, and prognosis of lung cancers. 

## Figures and Tables

**Table 1:** Immunohistochemical profiles of squamous cell carcinoma and pulmonary adenocarcinoma.

Special stain/IHC	Squamous cell carcinoma	Adenocarcinoma
CK7	−/+	+
p63	+	−
CK5/6	+	−
TTF-1	−	+/−

**Table 2:** Immunohistochemical profile of neuroendocrine tumors.

IHC	Typical carcinoid	Atypical carcinoid	Primary lung small cell ca.	Metastatic small cell ca.
CD56	+	+	+	+
Synaptophysin	+	+	+/−	+/−
Chromogranin	+	+	−/+	−/+
TTF-1	−/+	−/+	Usually +	Usually −
Ki-67	0–5%	5–15%	>20% (typically >60%)	>20% (typically >60%)

**Table 3:** Clinically relevant molecular and clinicopathologic features of NSCLC.

	EGFR mutation	K*-ras* mutation	EML4-ALK mutation
Histologic subtype	Adenocarcinoma	NSCLC (Adeno > squamous)	Adenocarcinoma, signet ring morphology
Clinical features	Asian, female, light/never smokers	Past or present smokers	Light/never smokers, possible young age and male
Significance	Sensitive to EGFR TKI therapy	Relative resistance to EGFR TKI therapy, possible poor prognostic marker	Use of ALK inhibitors (currently under study)
